# Upper Limb Muscle Activity among Workers in Large-Herd Industrialized Dairy Operations

**DOI:** 10.3389/fpubh.2016.00134

**Published:** 2016-06-28

**Authors:** Anthony Mixco, Federica Masci, Colleen Annika Brents, John Rosecrance

**Affiliations:** ^1^Department of Environmental and Radiological Health Sciences, Colorado State University, Fort Collins, CO, USA; ^2^Department of Health Sciences, International Centre for Rural Health, San Paolo Hospital, University of Milan, Milan, Italy; ^3^Department of Environmental and Occupational Health, Colorado School of Public Health, Aurora, CO, USA

**Keywords:** ergonomics, dairy workers, milking, surface electromyography, work-related musculoskeletal disorders

## Abstract

**Objectives:**

The primary aim of this cross-sectional research study was to quantify upper limb muscle activity among workers performing milking tasks in large-herd dairy parlors.

**Methods:**

Surface electromyography (sEMG) from the trapezius, anterior deltoid, biceps brachii, wrist flexors, and wrist extensors muscles of 26 dairy workers were used to create muscle activity profiles for the milking tasks common in large-herd dairy parlors. Functional maximum voluntary contractions (fMVC) were collected to normalize the sEMG data for appropriate comparisons. Anthropometric measurements were recorded from each worker.

**Results:**

The biceps brachii had the highest muscle activity (14.58% fMVC) of the upper limb muscles measured, exceeding previously established recommendations for working tasks. The anterior deltoid had the least amount of activity, while the upper trapezius had the least amount of muscular rest during milking work. Worker stature was negatively associated with upper limb muscle activity.

**Conclusion:**

Milking tasks in large-herd dairy parlors have significant effects on the upper limb muscle activity of workers. The muscle activity of biceps brachii during normal work tasks exceeded the recommended safe limit. Wrist flexors and upper trapezius approached the recommended limit. The study findings suggest that milking tasks in large-herd dairies may increase the worker’s risk for developing musculoskeletal symptoms and possibly musculoskeletal disorders.

## Introduction

Dairy farming is one of the oldest agriculture practices in human history (1). Throughout the last 200 years, modern milking operations have drastically changed from their ancestral counterparts in both size and technology used. What were once considered large farms of 20–25 cows using manual foot-powered Mehring milking machines in the 1890s have become operations of 1500+ cows with parlor milking systems (2). Advances in milking technology combined with economics of scale have led to the industrialization of the modern dairy farm. Large- and mega-herd dairy farms in the U.S. consist of 5% of American dairy operations but produce 65% of the domestic milk (3). Despite the economic advantages of large-herd milking operations, the industrialization of dairy processes has led to highly repetitive and physical work demands, which have been associated with the development of musculoskeletal disorders (MSDs) (4, 5).

To date, occupational health research within the dairy industry has primarily been focused on workers employed on small-herd farms (6–13). Occupational health researchers have concluded that milking tasks on small-herd farms require high muscular load (11, 13), consist of highly repetitive motions (6, 12), are physically demanding (14), and are associated with musculoskeletal symptoms (MSS) (8, 15) and MSDs (6, 11, 15). The same occupational risk factors identified in small-herd dairies can be expected in large-herd dairy farms; perhaps, to an even greater extent since they require employees to perform the same highly repetitive milking tasks for 812 h per work shift, 6–7 days a week. Researchers have suggested that work performed with large-herd parlor systems increase the risk of injury (16, 17). Yet, industrialized operations have not been well-studied and relatively little is known about the precise muscle loads, duration of muscle use, and muscle fatigue among the parlor workers.

The primary aim of the present cross-sectional research study was to quantify upper limb muscle activity of workers performing milking tasks in large-herd dairies. A secondary aim was to investigate associations between anthropometric variables and surface electromyography (sEMG) activity recorded during milking tasks. Surface EMG from the trapezius, anterior deltoid, biceps brachii, wrist flexors, and wrist extensors muscles was used to create muscle activity profiles for the combination of milking tasks performed in large-herd dairies. This is the first published study that has quantified muscle activity with sEMG of the upper limb at large-herd U.S. dairy operations.

## Materials and Methods

### Participants

Based on sample size calculations (see Statistical Analyses) and oversampling, we intended to recruit up to 30 workers (26 based on power calculations plus oversampling of 4). Dairy parlor workers were recruited from a pool of 36 dairy parlor workers employed at six large-herd dairy farms in Colorado, USA. Inclusion criteria included 18 years and older, free from any current musculoskeletal pain, and at least 6 months of experience working in a dairy parlor. Recruitment of workers was conducted through verbal announcements at the dairy and by paper notices posted in the indoor lunch area of the dairies. Subjects were compensated $30 for their participation. This study was carried out in accordance with the recommendations of Institutional Review Board of the investigator’s university, with written informed consent from all subjects. All subjects (including dairy company owners) gave written informed consent in accordance with the Declaration of Helsinki.

### Data Collection Procedures

**Figure 1:**
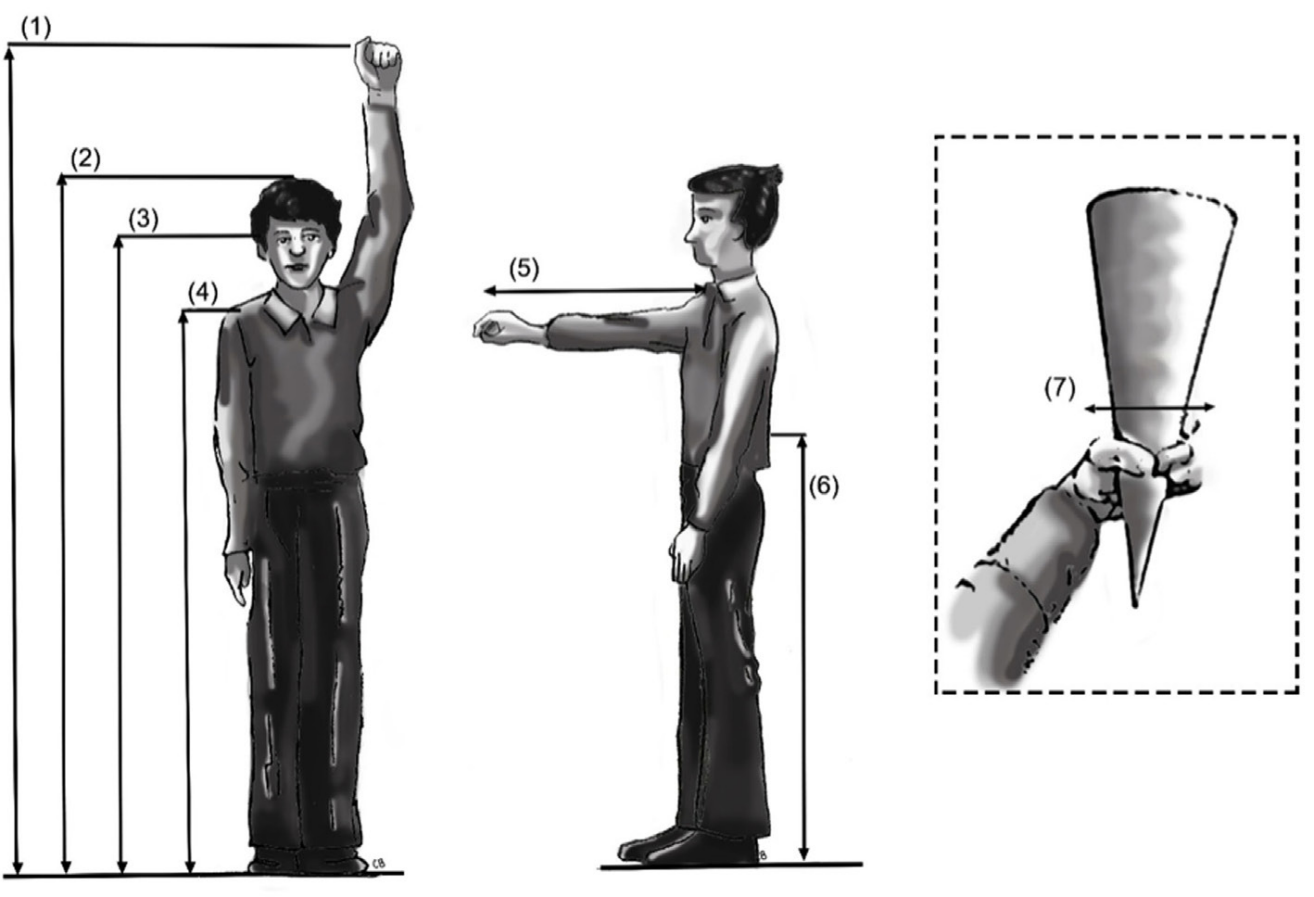
**Anthropometric measurements recorded (1) functional overhead reach, (2) standing height, (3) eye level height, (4) shoulder acromial height, (5) functional forward reach, (6) waist height, and (7) grip breadth**.

Several anthropometric measurements were recorded from each worker as illustrated in Figure 1. These measurements included functional overhead reach, standing height, standing height wearing boots, eye level and shoulder acromial height, forward functional reach, waist height, and grip breadth, which was measured as the circumference between the thumb and middle finger.

Surface electromyography with a sampling frequency of 1000 Hz using Biometrics DataLOG (Biometrics, England) was collected from the upper trapezius, anterior deltoid, biceps brachii, wrist flexors, and wrist extensors. Bipolar electrodes (Biometrics, Ltd.) were attached to skin with double-sided tape directly over the midsection of the muscle belly of the dominant arm. The appropriate sEMG electrode placement was determined by palpitation while functional movements of the upper limb were performed (18). Real-time streaming of sEMG was visually examined to assure that muscle activity coincided with appropriate functional movements (e.g., raising and lowering the upper limb to check activation of the anterior deltoid).

### Functional Maximum Voluntary Contraction Procedures

Functional maximum voluntary contractions (fMVC) were collected to normalize sEMG data for appropriate comparison. Before commencing fMVC, a 30-s baseline resting sEMG signal was collected to establish a minimum resting muscle activity. Three fMVC trials were administered for each subject for each muscle group. Participants were instructed to ramp up to a maximum muscular effort, hold for 4 s. After each trial, a maximum was calculated using the middle 3 s of the root mean square (RMS)-processed sEMG trial data. Covariance was calculated using the mean and SD. If the covariance exceeded 15% for the three fMVC trials, additional trials were conducted up to a total of five.

Functional MVCs for the anterior deltoid and upper trapezius were collected using procedures established by Boettcher et al. (19). Wrist flexor and extensor fMVCs were obtained simultaneously through a co-contraction while gripping a hand dynamometer (Biometrics G100, England). Participants were instructed to maintain a maximum power grip on the dynamometer while maintaining the elbow in 90° of flexion. Functional MVCs for the biceps brachii were recorded simultaneously during the MVC procedures for the wrist flexor and extensors.

### Milking Tasks

All subjects completed the five distinct milking tasks common in large-herd dairy parlors, as follows: (1) pre-dipping the teats into a sanitizing solution, (2) stripping each teat to stimulate milk letdown, (3) wiping the teats to remove the sanitizing solution, (4) attaching the milking cluster, and (5) post-dipping the teats with a sanitizing solution. Typically, pre-dipping, wiping, and post-dipping were completed with one arm, while stripping and attaching required both arms/hands. With tasks that could be completed using one arm, workers were instructed to use the arm instrumented with sEMG (same side as dominant hand). To develop muscle activity profiles, it was necessary to precisely determine when milking tasks began and ended. This was accomplished using a digital-event-marker that was triggered within the sEMG stream at the start of the data collection period. Data were collected on each worker for the duration of time it took to completely milk one pen of cows (typically 225–275 cows), which ranged from a 45- to 90-min period. During the data collection period, there were no breaks (bathroom, smoking, lunch, etc.) and little time, if any, for other tasks. If other tasks were performed, they were related to the milking tasks, such as refilling the towel dispenser, hosing off the stall with water, or refilling the supply of teat cleansing solution. Any short periods of rest, as well as periods involving other minor work tasks, were collected in the sEMG sample. The authors estimate that the other minor milking tasks involved less than 5% of the workers time during the actual data collection period.

### Muscle Activity Profiles

Following the normalization of all sEMG data using functional MVCs, muscle activity profiles were developed. Functional MVC data were processed with 100 ms moving average as recommended in the literature (20). The maximum value determined from this processing procedure was used to normalize the sEMG data collected during the milking tasks. The sEMG data were normalized using the instantaneous maximum value determined from the highest 100 ms average. Normalization was completed using an arithmetic process, where %*MVC* is normalized muscle activity, *sEMG* represents processed sEMG data, *fMax* represents instantaneous maximum value from fMVC trials, and *Rest* represents the minimum value from the 30-s baseline.

(1) $$\%\text{MVC}=\frac{\left(\text{sEMG}-\text{Rest}\right)}{\left(\text{fMax}-\text{Rest}\right)}$$

Temporal analysis of sEMG data was accomplished through the RMS processing technique (21). A graphic user interface was created using MATLAB 7.10.0 (Mathworks, Natick, MA, USA) to process sEMG data and obtain mean RMS values. Amplitude probability distribution function (APDF) was determined for the 10th, 50th, and 90th percentile (22) using custom software (21) developed in LabVIEW (National Instruments, Austin, TX, USA). Percentage muscular rest (%MR) of sEMG was determined with a maximum threshold of 0.5% MVC and a minimum gap duration of 0.25 s (23). The same LabVIEW custom software (21) was used to determine %MR values. Muscle activity profiles were constructed for each muscle with normalized muscle activity expressed as RMS, ADPF, and %MR. The muscle activity data were averaged across subjects for each muscle providing an estimate of the overall muscle activity and recovery experienced by parlor workers during the five milking tasks.

### Statistical Analyses

All statistical analyses were administered using SAS 9.3 (SAS Institute Inc., Cary, NC, USA). Sample size was determined from power calculations using a conventional alpha level of 0.05, a beta level of 0.20, representing 80% power, and effect magnitudes based on previously published EMG data from field (6, 12) and laboratory-based (24) studies. Descriptive statistics for subjects and muscle activity profiles were constructed. Muscle profiles were examined using a random block 26 × 5 ANOVA (Subject × Muscle) with a Tukey Honest Significant Difference *post hoc* adjustment to determine significant differences in the RMS, APDF, and %MR variables. Correlations among these three measures were also examined. Statistical significance was set at *p* < 0.05 *a priori*.

## Results

### Participants

Twenty-nine dairy parlor workers out of a possible 36 met the inclusion criteria and agreed to participate in the study. Two workers in the study were excluded from the data analysis due to equipment malfunctions that resulted in incomplete data. An additional subject did not show up on their scheduled day of sEMG collection and did not complete the study. Of the 26 participants whose data were analyzed, 25 were males and one was female. Workers were 18–53 years of age (mean = 29.7 ± 9.8 years) with work experience in cow dairies from 6 months to 20 years (mean = 3.4 ± 4.8 years). All participants had experience in the common milking tasks of pre-dipping, stripping, wiping, attaching milking clusters, and post dipping. The participants had an average functional stature of 166.4 cm (±9.3), an average forward reach of 61.3 cm (±3.5), and an average BMI of 26.4 (±4.2). Twenty-five of the participants were right-hand dominant, and one was left-hand dominant. Fifteen indicated at least some high school education and remaining participants indicated eighth grade or lower as their education level. All workers self-identified as Latino/a, with the majority from Mexico (*N* = 10) and Guatemala (*N* = 10). Latino workers account for the majority of the workforce in Colorado large-herd dairy parlors, as indicated by Patil et al. (14).

### Muscle Activity Profiles

**Figure 2:**
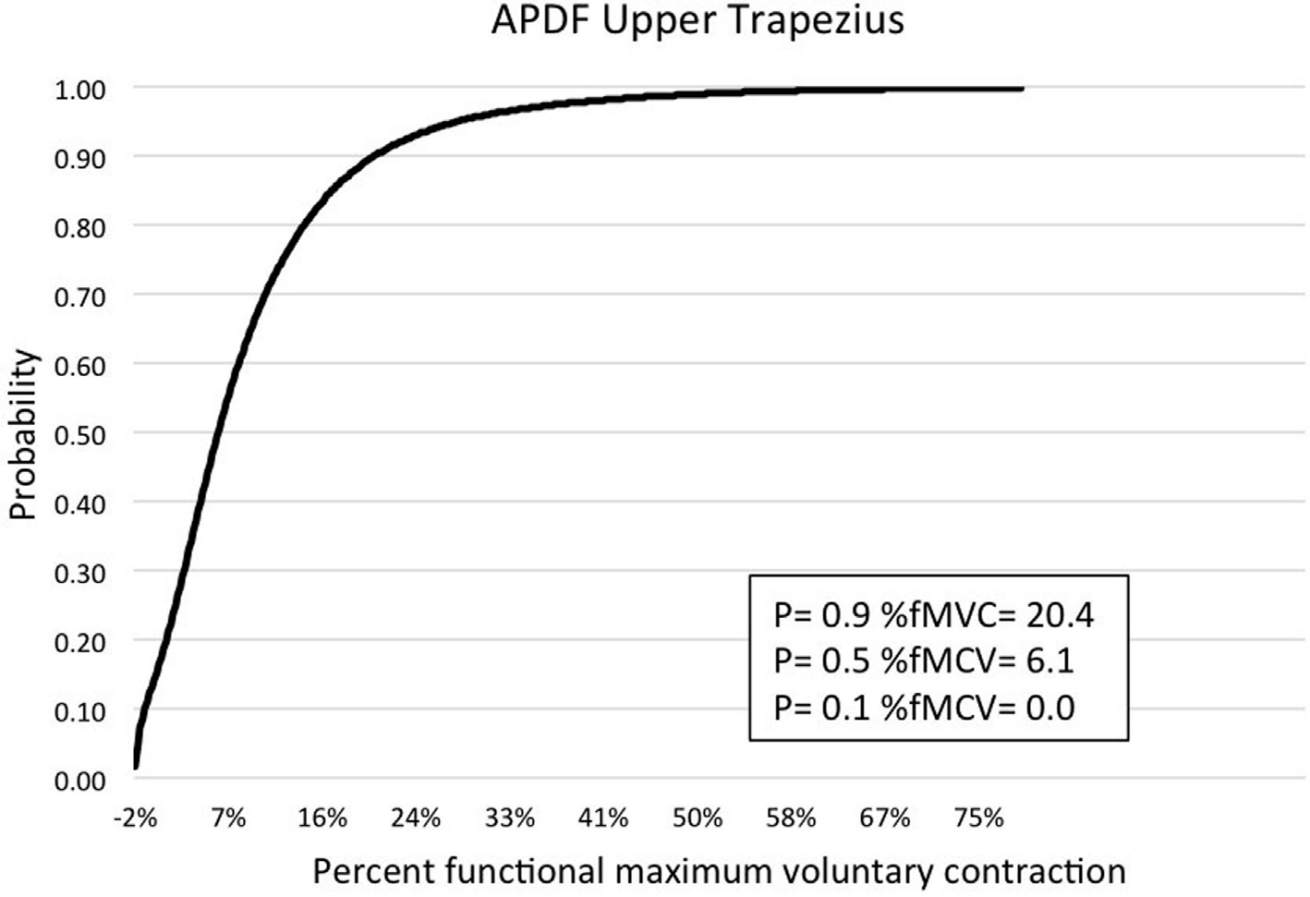
**Graphic representation of typical APDF of the upper trapezius**.

**Figure 3:**
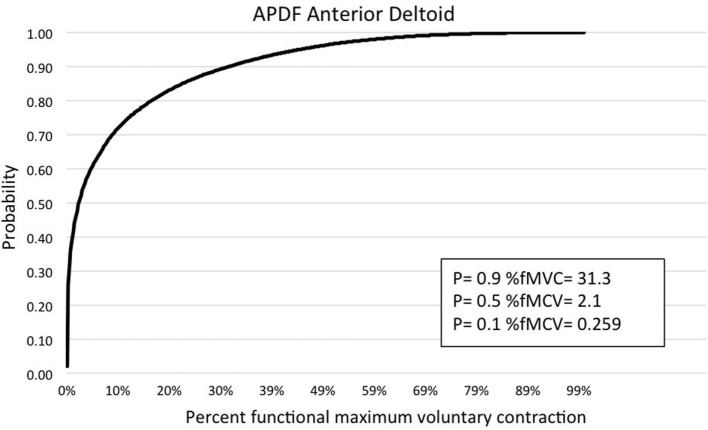
**Graphic representation of typical APDF of the anterior deltoid**.

**Figure 4:**
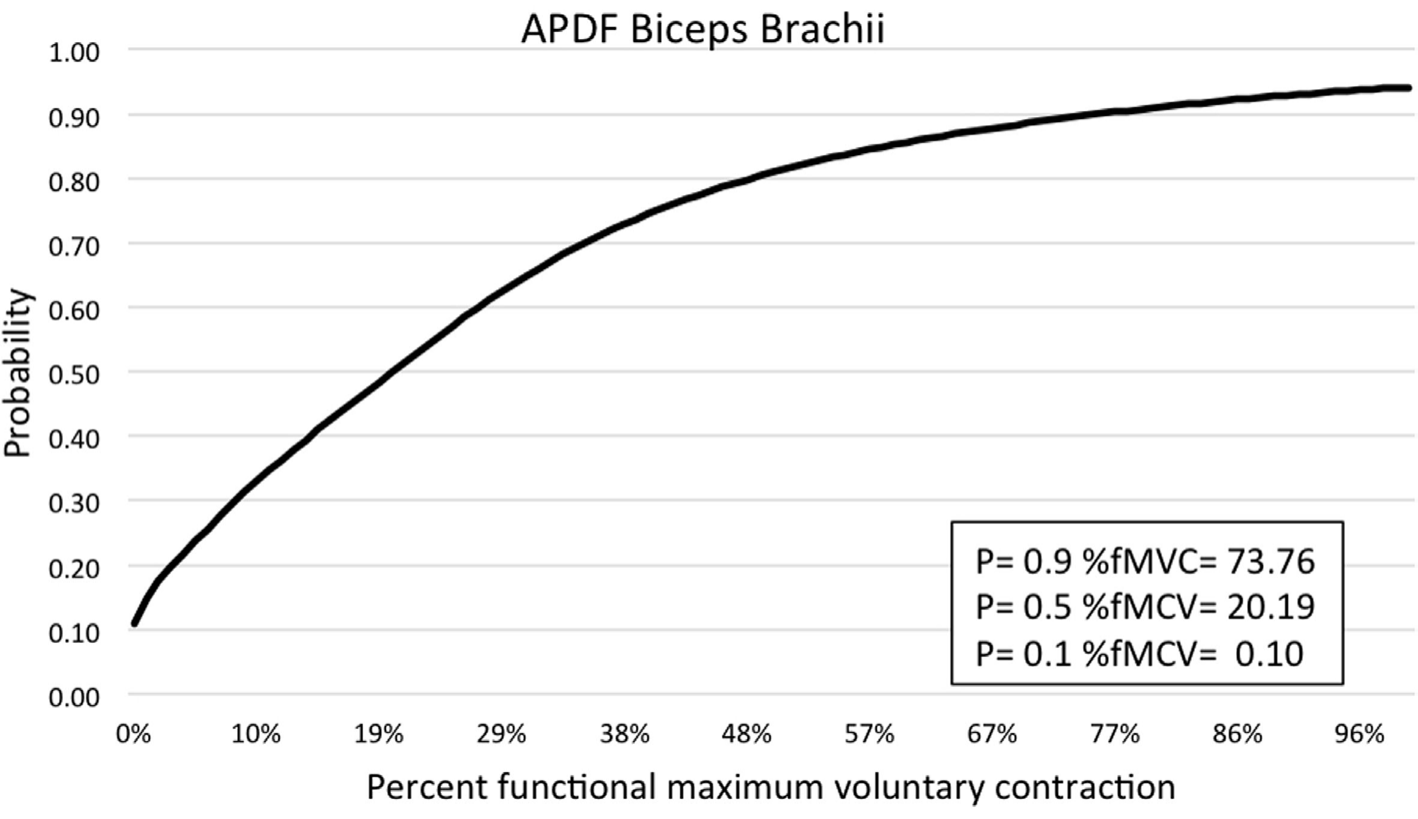
**Graphic representation of typical APDF of the biceps brachii**.

**Figure 5:**
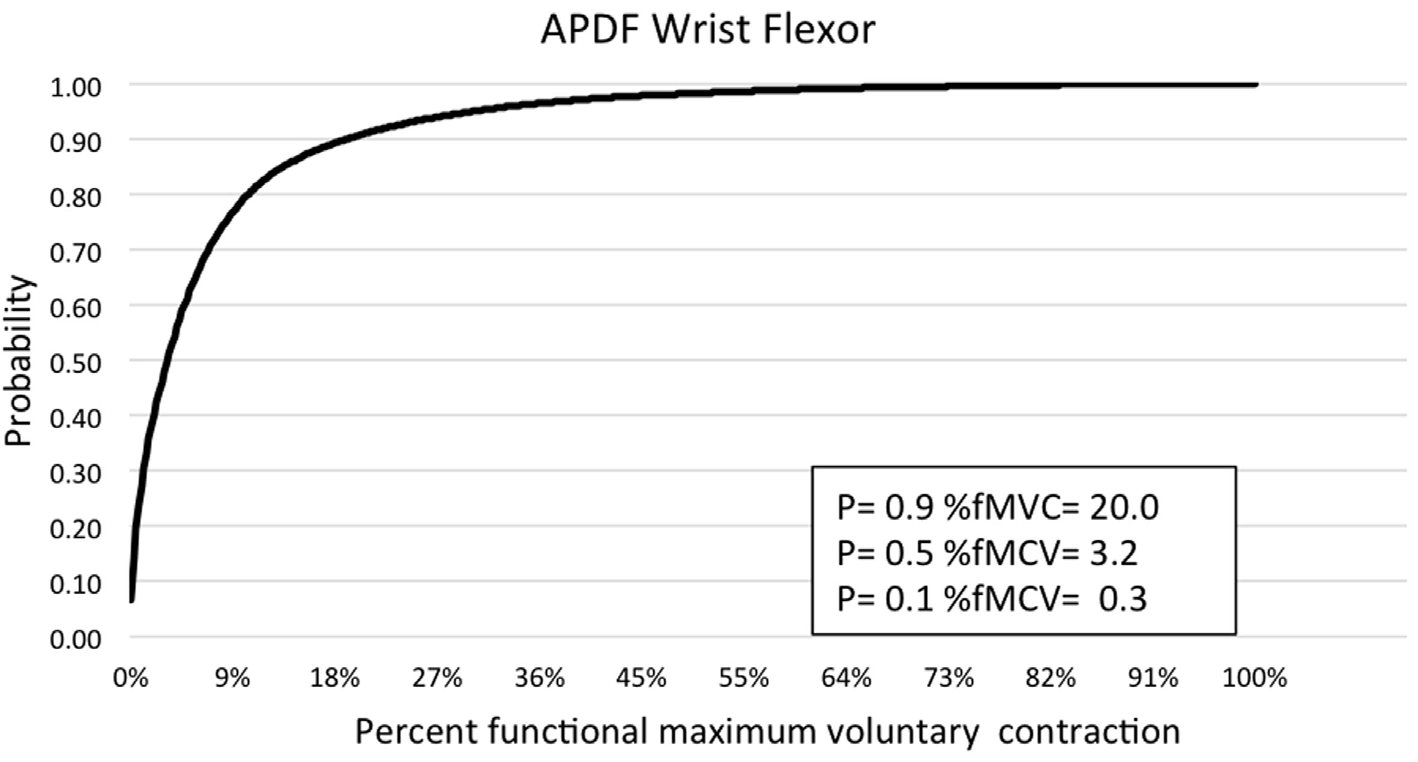
**Graphic representation of typical APDF of the wrist flexors in the forearm**.

**Figure 6:**
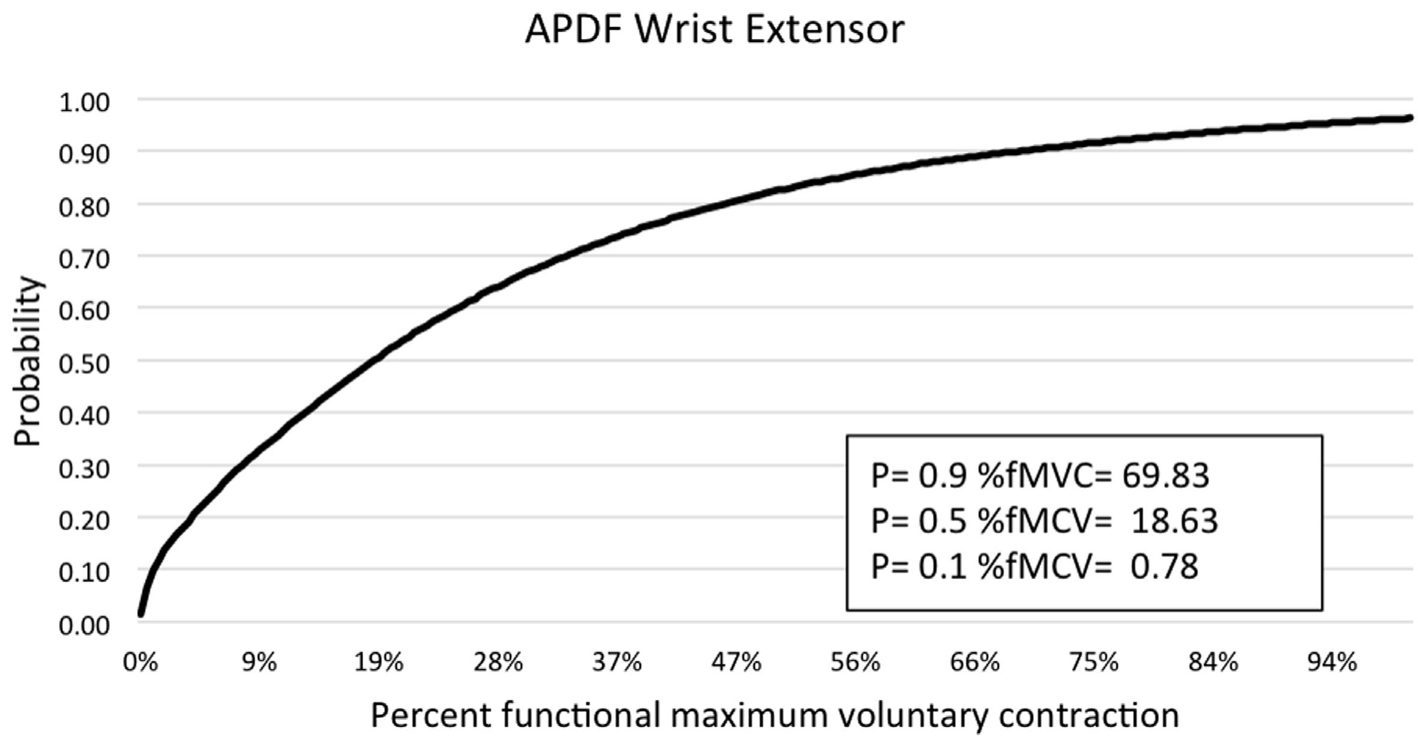
**Graphic representation of typical APDF of the wrist extensors in the forearm**.

Muscle activity profile data are outlined for the upper trapezius, anterior deltoid, biceps brachii, wrist flexors, and wrist extensors (Table 1). Additionally, muscle activity profiles of each muscle were constructed based on the APDF and are illustrated in Figures 2–6.

**Table 1:** **Activity profiles of upper extremity muscles studied**.

	Upper trapezius Mean (SD)	Anterior deltoid Mean (SD)	Biceps brachii Mean (SD)	Wrist flexors Mean (SD)	Wrist extensors Mean (SD)
10th percentile APDF	1.13 (2.07)	0.15 (0.39)	1.21 (2.23)	0.40 (0.60)	0.55 (0.92)
50th percentile APDF	9.28 (6.61)	3.49 (3.71)	14.58 (11.5)	7.41 (5.10)	9.75 (5.70)
90th percentile APDF	31.43 (21.05)	43.37 (36.26)	51.23 (38.86)	36.75 (21.41)	44.11 (31.13)
Mean RMS %fMVC	13.58 (9.19)	9.79 (3.71)	19.44 (13.87)	12.73 (6.24)	14.02 (7.73)
%MR	6.64 (7.24)	22.77 (12.75)	9.45 (7.73)	13.58 (8.25)	13.16 (6.69)

*RMS, root mean square; APDF, amplitude probability distribution function; %fMVC, percent functional maximum voluntary contraction; %MR, percent muscular rest*.

The ANOVA of the mean RMS indicated significant (*p* = 0.001) differences between anterior deltoid and biceps brachii (Table 2) with the biceps brachii having twice the mean activity of the anterior deltoid. Additionally, mean EMG activity for the wrist flexors were significantly (*p* = 0.05) less than biceps brachii. There were no other significant differences in the RMS muscle activity profiles.

**Table 2:** **ANOVA of mean RMS muscle activity for muscle pairs**.

Muscle	%fMVC mean (SD)	Delta	Adjusted *p*-value
Anterior deltoid vs. biceps brachii	9.79 (3.71) vs. 19.44 (13.87)	9.70	0.001
Wrist flexors vs. biceps brachii	12.73 (6.24) vs. 19.44 (13.87)	6.71	0.05

*RMS, root mean square; %MR; percent muscular rest; %fMVC, percent functional maximum voluntary contraction*.

Results of the normalized 50th percentile APDF ANOVA (Table 3) indicated that the anterior deltoid had significantly less muscle activity than biceps brachii (*p* < 0.001), upper trapezius (*p* = 0.03), and wrist extensors (*p* = 0.01), but not less than wrist flexors (*p* > 0.05) muscles during the milking tasks. Biceps brachii had significantly more muscle activity than wrist flexors (*p* = 0.003) and upper trapezius (*p* = 0.05).

**Table 3:** **ANOVA of 50th percentile APDF for muscle pairs**.

Muscle	%fMVC mean (SD)	Delta	Adjusted *p*-value
Anterior deltoid vs. biceps brachii	3.49 (3.71) vs. 14.58 (11.5)	11.08	<0.0001
Anterior deltoid vs. upper trapezius	3.49 (3.71) vs. 9.28 (6.61)	5.78	0.03
Anterior deltoid vs. wrist extensors	3.49 (3.71) vs. 9.75 (5.70)	6.25	0.01
Biceps brachii vs. wrist flexors	14.58 (11.5) vs. 7.41 (5.10)	7.18	0.003
Biceps brachii vs. upper trapezius	14.58 (11.5) vs. 9.28 (6.61)	5.30	0.05

*APDF, amplitude probability distribution function; %fMVC, percent functional maximum voluntary contraction*.

The ANOVA for normalized %MR (Table 4) indicated that the anterior deltoid had significantly greater rest than biceps brachii (*p* < 0.001), upper trapezius (*p* < 0.001), wrist extensors (*p* = 0.003), but not wrist flexors (*p* = 0.11) muscles. The upper trapezius had significantly (*p* = 0.02) less rest than wrist flexors.

**Table 4:** **ANOVA of %MR for muscle pairs**.

Muscle	%fMVC mean (SD)	Delta	Adjusted *p*-value
Anterior deltoid vs. biceps brachii	22.77 (12.75) vs. 9.45 (7.73)	13.32	<0.0001
Anterior deltoid vs. upper trapezius	22.77 (12.75) vs. 6.64 (7.24)	16.13	<0.0001
Anterior deltoid vs. wrist extensors	22.77 (12.75) vs. 13.16 (6.69)	9.61	0.003
Upper trapezius vs. wrist flexors	6.64 (7.24) vs. 13.58 (8.25)	6.94	0.02

*%MR; percent muscular rest; %fMVC, percent functional maximum voluntary contraction*.

### Anthropometric Analysis

Functional stature, shoulder acromial height, functional forward reach, hand grip breadth, BMI, and age were examined to determine if these anthropometric variables would be correlated with normalized mean RMS activity, APDF, and %MR. Combining data for all the muscles revealed significant negative correlations between mean RMS activity with functional stature (*R* = −0.22, *p* = 0.01) and mean RMS activity with shoulder acromial height (*R* = −0.20, *p* = 0.02). Thus, as worker’s height decreased, upper limb muscle activity tended to increase. Normalized APDF had similar statistically significant negative correlations with functional stature (*R* = −0.19, *p* = 0.01) and shoulder acromial height (*R* = −0.17, *p* = 0.01). Normalized %MR had significant positive correlations with functional stature (*R* = 0.23, *p* = 0.01) and shoulder acromial height (*R* = 0.22, *p* = 0.01). There were no other significant correlations between anthropometric variables and normalized RMS, APDF, and %MR. The correlations between functional stature and muscle activity measures indicated that shorter workers had more upper limb muscle activity and less muscular rest compared to taller workers.

Functional stature was examined to determine if there was an interaction effect with specific muscles for normalized mean RMS activity and %APDF. The normalized RMS ANOVA revealed no significant interaction between muscle and functional stature (*p* = 0.38). However, the main effects indicated that functional stature was significantly (*p* = 0.009) associated with mean RMS activity. The normalized APDF ANOVA also revealed no significant (*p* = 0.49) interaction between muscle and functional stature. Additionally, the main effects revealed functional stature as significantly (*p* = 0.02) associated with APDF.

Functional stature was also assessed to determine if an interaction occurred with muscle type for the normalized %MR. There was no statistically significant interaction between muscle type and functional stature; however, the interaction approached significance (*p* = 0.07). This finding suggests an interaction between muscle type and functional stature for %MR, which may be revealed in studies with larger sample sizes.

## Discussion

The aim of this research was to quantify upper limb muscle activity of workers performing milking tasks in large-herd dairies. Normalized mean RMS sEMG activity was significantly different between the biceps brachii and anterior deltoid muscles, which had the highest and lowest values, respectively. Normalized 50th percentile APDF revealed nearly identical results with the anterior deltoid displaying the least amount of muscle activity and biceps brachii displaying the highest mean muscle activity. These results were contrary to our initial hypothesis that the anterior deltoid would have the greatest amount of muscle activity during milking tasks. There were several possible explanations that could account for the anterior deltoid’s relatively low muscle activity during milking tasks. First, the middle deltoid also contributes to shoulder elevation but was not measured in the present study. Second, tasks performed near the worker’s shoulder height can be accomplished with minimal shoulder flexion through primarily elbow flexion if the task is performed close to the worker (minimal horizontal distance between worker and task). Additionally, the anterior deltoid may have been assisted in shoulder flexion by the action of the biceps brachii that is also active in shoulder flexion. Lastly, although the present study did not examine the effects of fatigue, it is plausible that over time the workers’ anterior deltoid adapted to increasing work demands placed on the shoulder joint by increasing biceps activity.

The anterior deltoid was revealed to have the most muscular rest, while the upper trapezius and biceps brachii had the least. The differences in %MR between the anterior deltoid and the trapezius and biceps may have been related to work activities other than milking tasks during the data collection period, which were not distinguished in this study. Observationally, brief breaks existed between milking groups of cows being milked within herringbone and parallel parlor configurations. Depending on the dairy and parlor configuration, some workers rested when one group of cows was exiting and the next group was entering the parlor. In some dairies, the time between milking groups of cows consisted of workers rinsing floors, hosing down stalls, folding towels, and checking equipment. Regardless, workers generally kept their shoulders in near neutral positions during non-milking tasks. Most of the non-milking tasks were accomplished using elbow flexion (biceps activity) for elevating the hand and tools while minimizing anterior deltoid activity. This is one possible explanation for the differences recorded in muscle usage between the anterior deltoid and biceps brachii. The relatively low level of %MR recorded for the upper trapezius may have been due to the muscle being used often for elevation and upward rotation of the shoulder (25) during reaching activities. The upper trapezius would also be active when the arm is in a relatively neutral position, while the hands are used for carrying equipment or operating tools. Dairy tasks have been characterized as physically strenuous and demanding for the upper extremity (11, 15, 26–28), which can result in increased muscular tension in the upper trapezius.

Stål et al. (11) examined muscular load of the biceps, along with the flexor and extensor muscles of the forearms with sEMG within loose housing and tethering milking systems. The researchers used APDF and %MR to assess muscle activity. Although researchers did not statistically compare muscle activity, the authors indicated that visual examination of the sEMG signals revealed possible differences between muscles. The APDF of the biceps appeared to have nearly half the muscular activity as the forearm extensors and flexors, but there was no notable difference between muscles when viewing %MR. Pinzke et al. (6) also examined the muscular loads of biceps and forearm flexors associated with drying, pre-milking, and attaching in a loose-housing milking system. The authors reported that the biceps also had nearly half the activity as compared to the forearm fingers and flexors for each task except attachment. But, unlike Stål et al. (11), they reported that the biceps exhibited greater muscular rest. Although both previously mentioned studies employed the same methodology to determine muscular rest and APDF, Pinzke et al. (6) investigated each of the milking tasks separately, while Stål et al. (11), like the present study, examined the average muscle activity for the entire milking period.

Amplitude probability distribution function has been commonly used to assess the risk of developing MSDs due to work overload (22). Recommended muscle activity levels to reduce the risk of MSDs were developed for static work (10th percentile APDF), mean activity (50th percentile APDF), and maximum activity (90th percentile APDF). Static APDF activity is recommended to be below 2% MVC and to never exceed 5% MVC. The recommended level for the mean APDF activity is less than 10% MVC and to never exceed 14%. The recommendation for maximum APDF activity is less than 50% MVC and to never exceed 70% MVC (29). In the present study, biceps brachii had the mean APDF value at 14.58% MVC, exceeding the recommended high limit of 14%. The mean APDF for the wrist extensors and upper trapezius were below the 10% threshold at 9.75 and 9.28% MVC, respectively. The values presented in this present study differ from those determined by other researchers. Stål et al. (12) evaluated biceps brachii and wrist flexors activity during assisted and unassisted cluster attachment tasks. The researchers determined APDF for the 50th percentile of wrist flexor activity was 13% MVC and 6.1% MVC for biceps activity during milking cluster attachment. Another research group examining muscle activity at the 50th percentile APDF for milking tasks determined that biceps brachii activity ranged from 5.9 to 9.8% MVC, while wrist flexors activity ranged from 7.5 to 27% MVC (6). However, both studies above examined sEMG by each of the specific milking tasks. As in the present study, Stål et al. (11) examined sEMG of the upper limb for all milking tasks combined. They determined the 50th percentile APDF for the biceps brachii (3.9% MVC), wrist flexors (7.4% MVC), and extensors (8.5% MVC). Wrist flexor and extensor activity levels in the study by Stål et al. (11) were comparable to those reported in the present study. However, for biceps brachii at the 50th percentile APDF, the Stål group reported 3.9 vs. 14.58% MVC in the present study. This difference could be related to the type of work activity demands conducted in small- vs. large-herd dairies. Additionally, the majority of workers in the Stål et al. (11) study were females, as opposed to the majority being males in the present study.

Although there are no field studies that have examined muscle activity of the upper limb in large-herd dairies, several researchers (22, 24, 29, 30) recreated large-herd dairy milking tasks in a laboratory setting to conduct in-depth simulations of cluster attachments to investigate the effects of cluster weight reduction. Those researchers determined through sEMG that the attachment of a common 2.4 kg milking cluster imposed a considerable muscular load on upper limb muscles (22, 24). Reducing the mass of the milking cluster to 1.4 kg decreased mean muscle activity up to 20% (24). The muscle activity was not calculated using APDF but rather using the normalized integrated sEMG similar to RMS processing. For most upper limb muscles, the sEMG results during milking tasks reported by Jakob et al. (24) were comparable to our sEMG RMS processed findings (Table 1) with the exception of the anterior deltoid. The mean RMS anterior deltoid activity in the present study (9.738% MVC) was much lower than that reported by Jakob et al. (24) at 23.39% MVC. However, that difference is likely explained by the different methods and tasks examined. Jakob et al. (24) examined the attachment of the milking cluster, a task that requires worker’s arms at or above shoulder height in order to complete the task, while the muscle activity measurements in the present study comprised all milking tasks performed in the dairy parlor.

There is evidence in the literature of a relationship between lack of muscular rest and the development of shoulder disorders among occupational tasks involving the upper trapezius. Veiersted et al. (31) examined the relationship between muscle usage (activity and rest) and the development of trapezius myalgia through sEMG analysis. Data collection sessions comprised 10 min of sEMG sampling, while the subjects worked at their habitual work rhythm. The 50th and 90th percentiles of the APDF were used to describe muscular load, and muscular rest was defined by activity gaps under 0.5% MVC and durations of 0.2 s. The authors reported that as muscular rest increased by an additional gap per minute, the subjects’ risk of developing work-related MSDs decreased 6%. Subjects who did not experience MSD had gap rates greater than 10.8 gaps per minute or roughly 4% muscular rest. Hansson et al. (23) revisited the use of %MR to examine the sensitivity of the trapezius when comparing different work tasks by hospital cleaners and office workers. For repetitive work tasks, the sEMG activity at the 50th percentile APDF ranged from 3.6 to 8.1% APDF for the trapezius, less than those found for large-herd dairy parlor tasks. Muscular rest for the repetitive work tasks ranged from 1.1 to 13.4% of total work time. In the present study, muscular rest for upper trapezius during milking tasks was 6.6%, which was within the range presented for repetitive office and cleaning work tasks. Hansson et al. (23) found that %MR was a more precise measure than various APDF percentiles of the trapezius for comparison of work tasks. Although the development of trapezius myalgia has not been specifically evaluated in the dairy industry, the risk of such a disorder is likely present, as dairy work has some of the same movement and load characteristics as repetitive manufacturing tasks (13). Dairy parlor tasks work have been associated with MSS and WRMSDs (5, 8, 11, 14, 15). For example, in large-herd U.S. dairies, almost three-fourths of the milking workers had MSS in some anatomical region (8). Additionally, Kolstrup (15) used a modified version of the Standard Nordic Questionnaire and reported a high prevalence of MSDs among workers in shoulders, hands/wrist, and low back among workers in small-herd Swedish dairies. Patil et al. (14) examined the prevalence of carpal tunnel syndrome among workers in large-herd dairies and concluded that the prevalence of carpal tunnel syndrome was significantly higher among workers performing milking tasks than those in other areas of the dairy. Although the present study did not find that overall wrist flexors and extensors sEMG activity was beyond recommended levels for the 10th, 50th, and 90th APDF percentiles, wrist extensor activity was approaching the maximum threshold for 50th APDF and 90th APDF percentiles. It may be possible that sEMG activity associated with a specific milking task, rather than a variety of milking tasks (as in the present study), would reveal wrist flexor and extensor muscle activity beyond the recommended APDF thresholds and increasing risk for developing a WRMSD (e.g., carpal tunnel syndrome).

### Limitations

Surface EMG represents a fairly non-invasive source of information on the state of skeletal muscle activity. However, the application of sEMG in occupational field studies has limitations due to inherent problems associated with sEMG. Some of the variables that may affect the sEMG signal other than the actual muscle activity include electrode configuration, electrode placement and orientation, procedures for determining a functional MVC, cross talk from other muscles, movement artifact, muscle movement under the surface of the electrode, tissue impedance, and signal processing. It is important to emphasize that the muscle activity reported in the present study represents a combination of all five major milking tasks described as well as other minor tasks associated with the milking process. This study was limited to overall muscle activity of the upper limb and not focused on specific milking tasks. Additionally, the results of this study are limited to the workers at large-herd dairies in Colorado, USA, and caution should be exercised with extending these results to other populations of dairy parlor workers.

## Conclusion

Milking tasks in a large-herd dairy parlor have significant effects on upper limb muscle activity of workers. The muscle activity of biceps brachii measured in the present study exceeded the recommended ≤10% MVC for the 50th percentile APDF. Wrist flexors and upper trapezius were approaching the recommended ≤10% MVC threshold. The study findings suggest that milking tasks in large-herd dairies may increase the worker’s risk for developing MSS and possibly MSDs.

Although this investigation represents novel work on upper limb muscle activity among workers performing milking tasks in large-herd industrialized dairies, additional research is needed to for targeted interventions, which could reduce pain MSDs. Future studies should focus on determining how each of the specific milking tasks contributes to upper limb muscle load as well as the kinematics of the upper limb during the work tasks. Other possible future research includes the effects of muscular fatigue from dairy parlor work and comparison of the present study findings to small- and medium-sized herd dairies.

## Author Contributions

All authors (AM, FM, CB, JR) have (1) contributed substantially to the conception or design of the work and/or the acquisition, analysis, or interpretation of the data for the work, (2) participated in drafting the work or revising it critically for important intellectual content, (3) approved the final version to be published, and (4) agreed to be accountable for all aspects of the work in ensuring that questions related to the accuracy or integrity of any part of the work are appropriately investigated and resolved. JR was the local principal investigator and lead academic for this part of the grant award. He led the design of the study, providing expertise in sEMG and occupational biomechanics, and made a significant contribution to both the interpretation of data and the writing of the final paper. AM was the doctoral researcher who was significantly involved in the design of the study, led the collection and analysis of data, significantly contributed to the interpretation of data, and wrote the first draft of the paper. FM was the doctoral researcher that contributed to the design, data collection, data analysis, and also the editing of the paper. CB was the student researcher contributing to the interpretation of data and co-editing the various drafts as well as the final paper.

## Conflict of Interest Statement

The research was conducted in the absence of any commercial or financial relationships that could be construed as a potential conflict of interest.
